# DP-MaizeTrack: a software for tracking the number of maize plants and leaves information from UAV image

**DOI:** 10.3389/fpls.2025.1698847

**Published:** 2025-11-10

**Authors:** LongHao Chen, YingLun Li, ChuanYu Wang, Na Jiang, XinYu Guo

**Affiliations:** 1 College of Information Engineering, Capital Normal University, Beijing, China; 2 Information Technology Research Center, Beijing Academy of Agriculture and Forestry Sciences, Beijing, China; 3 Beijing Key Lab of Digital Plant, National Engineering Research Center for Information Technology in Agriculture, Beijing, China

**Keywords:** YOLOv8 improvement, UAV imagery, maize seedlings, object detection, multi-scale feature enhancement, loss function optimization

## Abstract

In modern agricultural production, accurate monitoring of maize growth and leaf counting is crucial for precision management and crop breeding optimization. Current UAV-based methods for detecting maize seedlings and leaves often face challenges in achieving high accuracy due to issues such as low spatial-resolution, complex field environments, variations in plant scale and orientation. To address these challenges, this study develops an integrated detection and visualization software, DP-MaizeTrack, which incorporates the DP-YOLOv8 model based on YOLOv8. The DP-YOLOv8 model integrates three key improvements. The Multi-Scale Feature Enhancement (MSFE) module improves detection accuracy across different scales. The Optimized Spatial Pyramid Pooling–Fast (OSPPF) module enhances feature extraction in diverse field conditions. Experimental results in single-plant detection show that the DP-YOLOv8 model outperforms the baseline YOLOv8 with improvements of 3.9% in Precision (95.1%), 4.1% in Recall (91.5%), and 4.0% in mAP50 (94.9%). The software also demonstrates good accuracy in the visualization results for single-plant and leaf detection tasks. Furthermore, DP-MaizeTrack not only automates the detection process but also integrates agricultural analysis tools, including region segmentation and data statistics, to support precision agricultural management and leaf-age analysis. The source code and models are available at https://github.com/clhclhc/project.

## 1 Introduction

With the continuous growth of the global population and the rising demand for food, achieving simultaneous improvements in crop yield and quality under limited arable land has become a pressing challenge for modern agricultural science (Yu and Wang, 2025). Maize (Zea mays L.), as one of the world’s three major staple crops, plays a pivotal role in ensuring food security and supporting agricultural economic development (Feng et al., 2025). Among the many factors affecting maize yield, seedling-stage population structure and individual traits—particularly plant density distribution and leaf development—directly determine subsequent photosynthetic efficiency, canopy architecture, and stress resistance (Jia et al., 2024). Therefore, the rapid and accurate acquisition of key phenotypic traits during the seedling stage is essential for optimizing planting density, improving resource-use efficiency, and advancing precision agriculture.

Traditional field-based phenotyping methods rely heavily on manual investigation, which is inefficient, subjective, and incapable of meeting the demand for large-scale, multi-site, and dynamic monitoring (Helia et al., 2025). In recent years, the rapid development of unmanned aerial vehicles (UAVs) and high-throughput phenotyping technologies has greatly expanded the application of low-altitude remote sensing in agriculture (Chang et al., 2023). UAVs are characterized by strong maneuverability, ease of operation, and wide coverage, enabling efficient acquisition of high-resolution RGB, multispectral, and thermal imagery for crop monitoring (Bo et al., 2021). Against this backdrop, deep learning–based object detection methods applied to UAV-derived RGB imagery have demonstrated strong potential in tasks such as seedling distribution mapping, canopy structure analysis, and leaf identification (Jia et al., 2023).

Nevertheless, achieving high-precision detection under complex field conditions remains challenging due to illumination variation, background interference, plant overlap, and morphological diversity. The YOLO (You Only Look Once) family of algorithms (Liu et al., 2025), known for its end-to-end design, high speed, and accuracy, has been widely adopted for agricultural vision tasks. However, the latest version, YOLOv8 (Cai et al., 2025), still suffers from insufficient robustness when applied to real-world field environments. Compared to industrial datasets, agricultural imagery often features large variations in object scale, dense occlusion, and complex backgrounds, leading to degraded detection performance. Moreover, (Islam et al., 2025) algorithmic improvements alone are insufficient to address practical needs; there is an urgent demand for an integrated software platform that not only incorporates detection but also provides data analysis and visualization capabilities, thereby enabling field-level deployment and delivering actionable insights for agricultural research and production.

Previous studies have attempted to enhance YOLO’s adaptability to agricultural scenarios through lightweight backbone networks (Liu and Li, 2025; Udeh et al., 2025), attention mechanisms (Yan et al., 2024; Zhang et al., 2025; Zhou et al., 2025), improved feature fusion structures (Liu et al., 2024; Wenming et al., 2024), and optimized regression loss functions (Joshi and Diwakar, 2024). However, most of these efforts have focused on single-object detection tasks such as fruit counting or disease spot recognition (Xie et al., 2025), while research on “maize seedling–leaf” dual-object detection remains limited (Pu et al., 2023) and insufficiently validated in real-world field environments (Yue and Zhang, 2025). More critically, these works remain confined to model-level experimentation and lack supporting software tools for agricultural application. Without an integrated software system, users cannot perform interactive management of detection results, regional segmentation, statistical analysis, or visualization, thereby constraining the scalability and applicability of such approaches in precision agriculture. Future research must therefore focus not only on algorithmic optimization of YOLOv8 but also on the development of practical software platforms that enable a complete pipeline from detection to application.

To this end, we developed an integrated detection and visualization system, DP-MaizeTrack, with two main contributions: (1) we propose a maize seedling detection model, DP-YOLOv8, specifically tailored for UAV remote sensing imagery of small objects, by incorporating a Multi-Scale Feature Enhancement (MSFE) module, an Optimized Spatial Pyramid Pooling Fast (OSPPF) module, and a dynamic IoU compression mechanism (Focaler-IoU), thereby significantly improving model performance under complex field conditions; and (2) we build a deployable software platform that embeds the improved detection model into an intuitive interface, enabling automated UAV image processing, visualization of detection results, and rapid leaf counting, thus bridging the gap from academic algorithm to practical tool. Collectively, this study advances both algorithmic performance and software applicability, providing a feasible solution for precision crop management and intelligent decision-making in agriculture.

## 2 Materials and methods

To facilitate efficient detection of maize seedlings and their leaves, this study conducted UAV-based image acquisition in a representative maize cultivation area of Yuanyang County, Henan Province. The region is characterized by flat terrain and concentrated maize planting, making it well-suited for UAV operations. Data collection was carried out during the seedling growth stage, when the morphological features of maize plants and leaves are most distinct and inter-plant occlusion is minimal, thus providing optimal conditions for training and validating detection models.

### 2.1 Data collection and dataset construction

**Figure 1:**
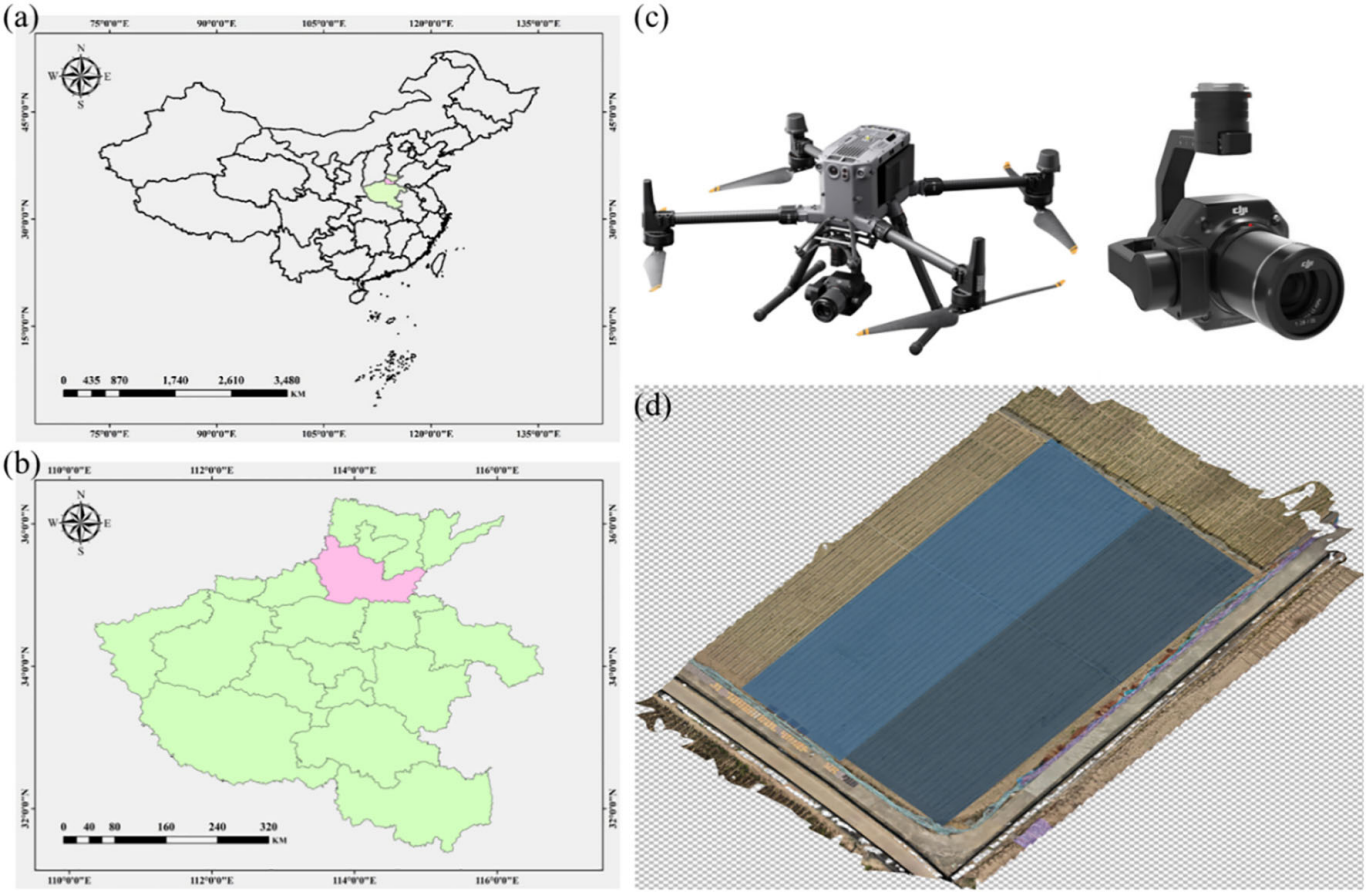
Experimental area, data-collection equipment, and original data illustration. Panels **(a, b)** show maize seedling data collection in Yuanyang County, Henan Province, China. Panel **(c)** presents the DJI UAV and high-resolution visible-light camera used for image acquisition. Panel **(d)** depicts the orthomosaic of the experimental field generated from UAV imagery, with the field subdivided into 728 plots representing 360 maize varieties.

Image acquisition was conducted using a high-resolution Zenmuse P1 UAV-mounted camera with high pixel density and minimal distortion, enabling clear capture of maize seedling details. Flight altitude and speed were carefully adjusted to ensure sufficient spatial resolution and coverage. The specific imaging parameters were as follows: Zenmuse P1 camera with a resolution of 8192 × 5460 pixels, 35 mm lens focal length, flight altitude of 20 m, and flight speed of 2 m s^-^¹. To minimize shadow interference caused by low solar elevation, all images were captured at 11:00 a.m. during the maize seedling stage.Plant-detection experiments were conducted directly on the original high-resolution images (8192 × 5460 pixels), as the overall plant structure was sufficiently salient to be clearly identified. Given the relatively simple plant morphology, spatial distribution, and limited background interference, no complex preprocessing was required. In contrast, leaf-detection experiments imposed greater demands: the collected images underwent a series of preprocessing steps to improve data quality and enhance training performance. Specifically, each image was cropped to remove superfluous background regions while retaining only the areas containing maize plants and leaves. The cropped patches were subsequently resized to a uniform resolution of 1024 × 1024 pixels to meet the input requirements of the YOLOv8 model. The study area and acquisition method are illustrated in **Figure 1**.

Data annotation is a pivotal step in object detection, as its quality directly determines the effectiveness of model training. In this study, manual annotation was conducted on pre-cropped images, including the location of each maize plant, the number of leaves, and the position of individual leaves. LabelImg was employed as the annotation tool owing to its simplicity and efficiency. All annotations were stored in YOLO format to facilitate subsequent training and validation. For plant detection, which uses high-resolution images, 50 annotated samples were sufficient for the experiments, whereas for leaf detection, conducted on lower-resolution images, 200 annotated samples were provided.To evaluate model performance, the annotated dataset was divided into training, validation, and test subsets in an 8:1:1 ratio. Specifically, 80% of the data were allocated for training, 10% for validation, and 10% for testing. This allocation ensured adequate data for training while enabling reliable performance assessment during validation and testing. Random sampling was employed to partition the dataset, ensuring uniform distribution and preventing performance bias due to uneven data representation.

Through these data acquisition and processing steps, we established a high-quality dataset for maize seedling and leaf detection, providing a solid foundation for subsequent model development. The dataset not only encompasses diverse morphological characteristics of maize seedlings at the seedling stage but also enhances diversity and usability through data augmentation and meticulous annotation, thereby offering strong support for efficient training and accurate model detection.

### 2.2 Model improvements

#### 2.2.1 DP-YOLOv8 model design

YOLOv8 is an efficient object detection model (Song and Li, 2025) that inherits the YOLO family’s strength in balancing real-time speed and detection accuracy. Its network architecture comprises three primary components (Qu et al., 2025): the Backbone, the Neck (feature-fusion module), and the Head (detection layer). The Backbone is responsible for extracting image features, the Neck fuses features across different hierarchical levels, and the Head performs final object detection and classification. Through an improved anchor mechanism and refined loss functions (Gong et al., 2025), YOLOv8 further enhances both detection accuracy and speed, delivering strong performance across diverse object detection tasks.

Despite its advantages, detecting maize plants and leaves in complex field environments remains challenging (Yu et al., 2025). Background clutter, variable illumination, plant occlusion, and large-scale leaf variation can all reduce detection accuracy. Therefore, task-specific improvements to YOLOv8 are essential for maize seedling and leaf detection.

**Figure 2:**
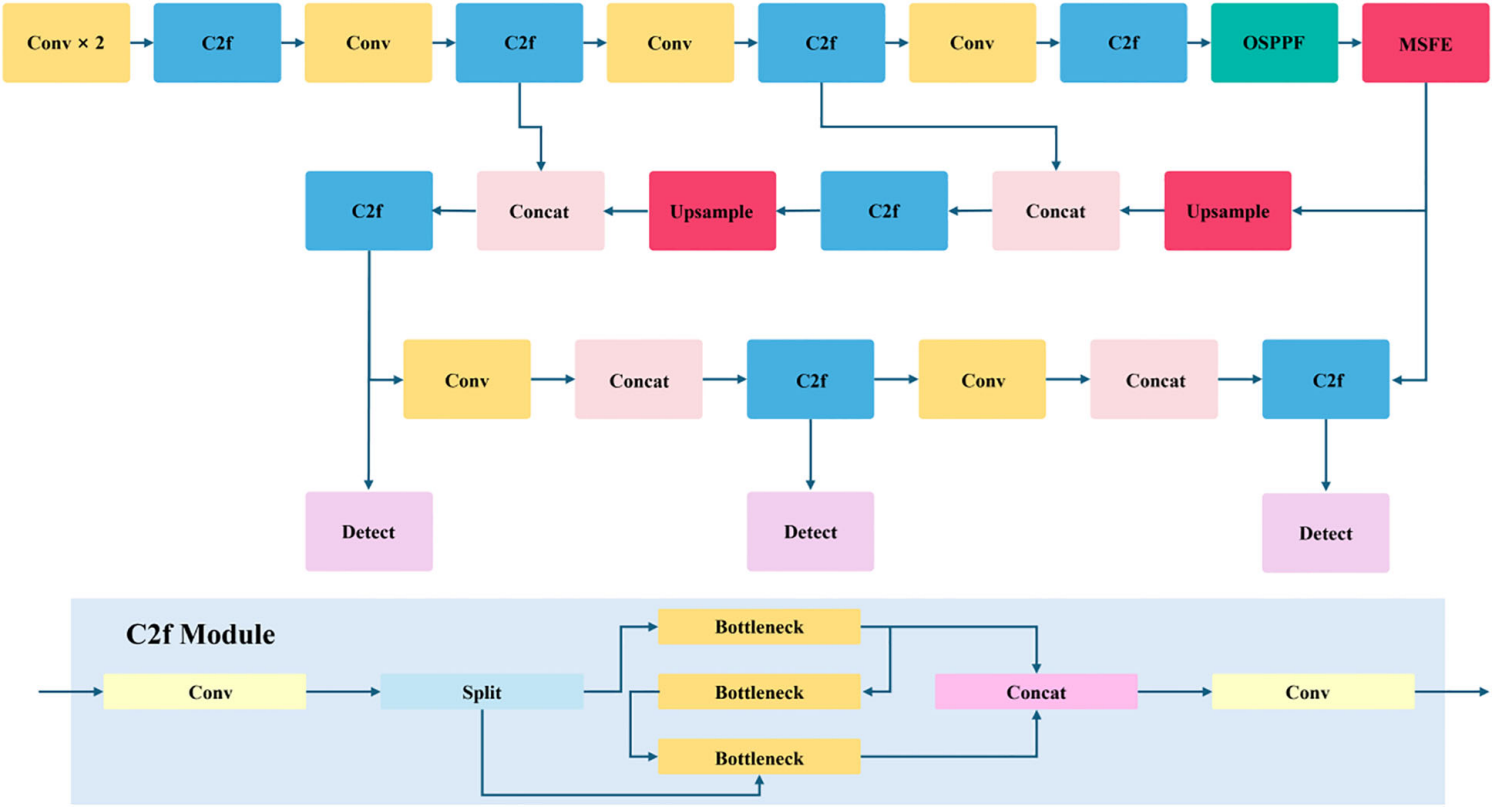
Architecture of the DP-YOLOv8 model for in-field maize plant detection. The network integrates two key enhancements: (i) incorporation of Global Average Pooling (GAP) and Global Max Pooling (GMP) branches into the SPPF module to strengthen multi-scale feature extraction, and (ii) introduction of the Multi-Scale Feature Enhancement (MSFE) module, which combines the ECA channel-attention mechanism with a multi-branch convolution structure to improve perception of fine-grained leaf details.

To address these challenges, we optimize the YOLOv8 Backbone by integrating a Multi-Scale Feature Enhancement (MSFE) module and an Optimized Spatial Pyramid Pooling–Fast (OSPPF) module. These modifications strengthen the model’s perception of multi-scale features, particularly for small objects and large-scale variations.To provide an intuitive overview of the improved YOLOv8 architecture, the overall network diagram is presented in **Figure 2**.

The figure illustrates the Backbone, Neck, and Head components and explicitly highlights the integration points of MSFE and OSPPF. With this structural design, the model is better equipped for maize plant and leaf detection in complex field environments.

#### 2.2.2 MSFE module

**Figure 3:**
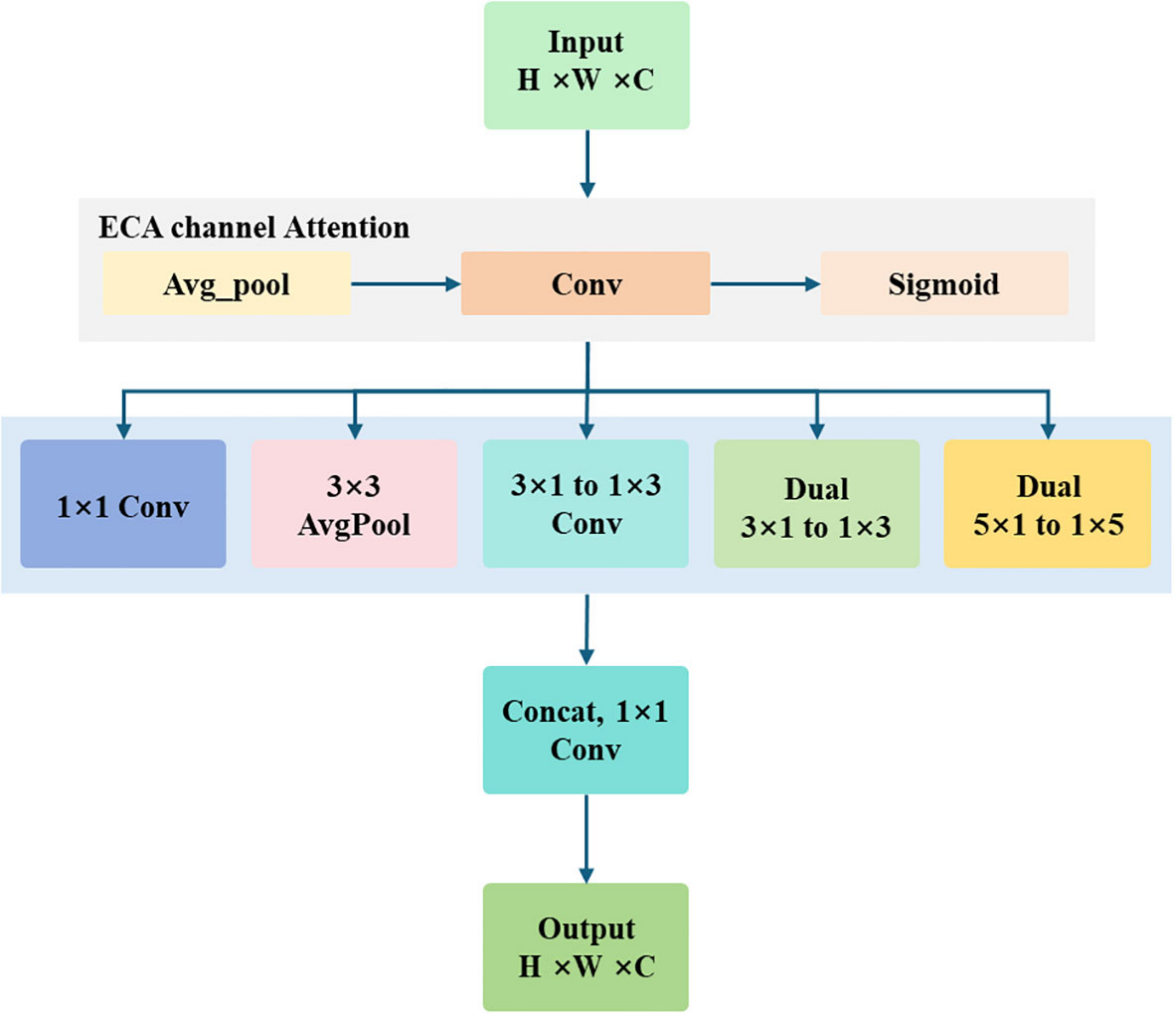
Structure of the Multi-Scale Feature Enhancement (MSFE) module. The module first applies the ECA channel-attention mechanism to adaptively recalibrate input features, and then employs three parallel branches to extract complementary information: a 1×1 convolution branch for channel compression and interaction, a 3×3 average-pooling branch for global context capture, and an asymmetric-convolution branch (cascaded 3×1 and 1×3 convolutions) for spatial feature extraction with reduced parameters. Finally, channel concatenation (Concat) integrates the outputs to achieve multi-scale feature fusion.

The MSFE module significantly improves multi-scale target perception by combining the Efficient Channel Attention (ECA) mechanism (Liu et al., 2025) with multi-branch feature extraction (Wang et al., 2025). ECA dynamically adjusts channel weights based on inter-channel relationships, thereby emphasizing salient features. Placing the ECA module before multi-branch extraction first filters the channels and then extracts features, effectively reducing redundant computation and improving efficiency. The multi-branch section employs sliding windows with different receptive fields to capture fine-grained leaf details, further enhancing the model’s ability to distinguish leaves. The structure of the MSFE module is illustrated in **Figure 3**.

#### 2.2.3 OSPPF module

**Figure 4:**
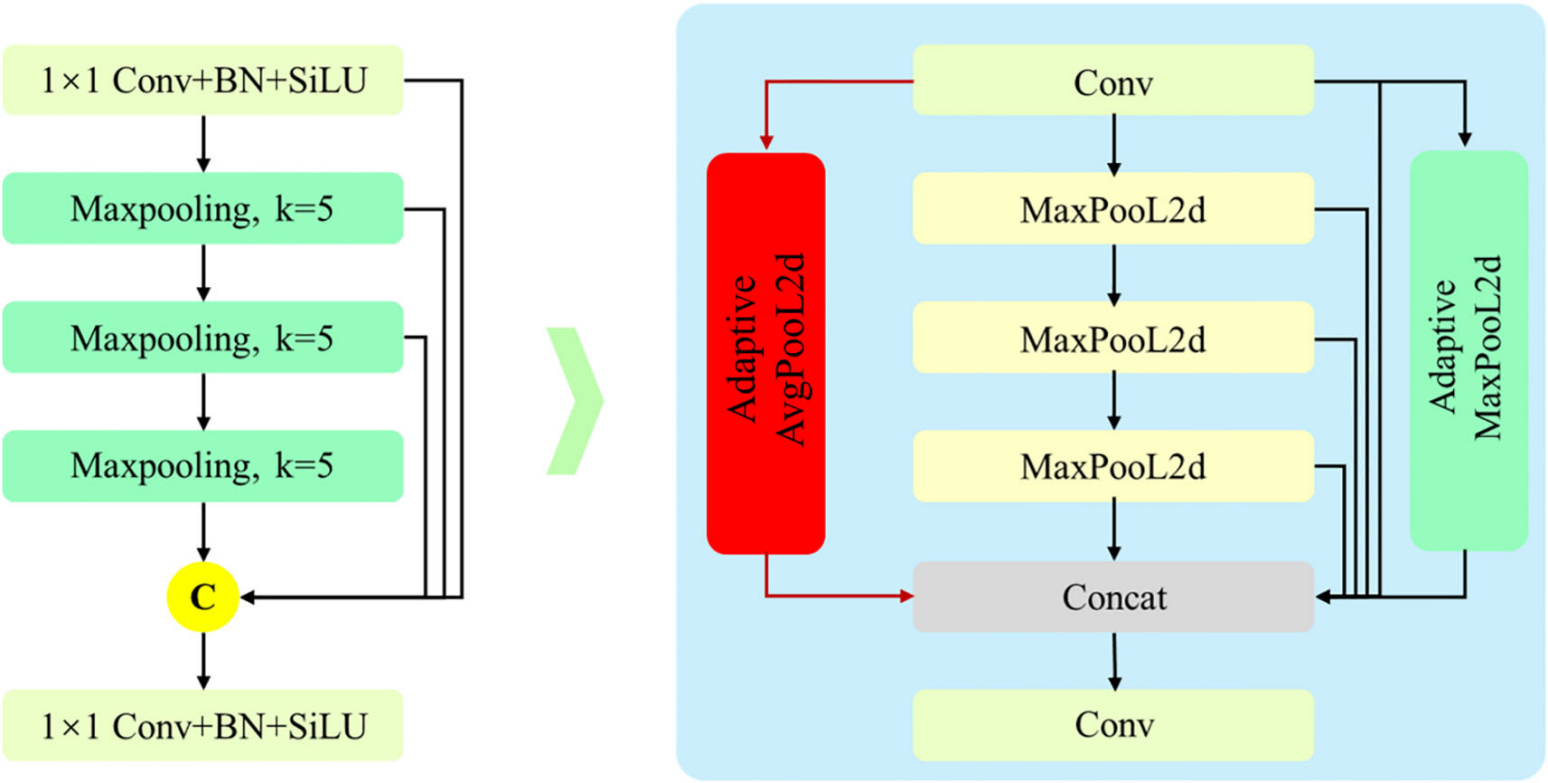
Structure of the optimized SPPF (OSPPF) module. Based on the original SPPF, an additional parallel branch is introduced at the input, incorporating Global Average Pooling (GAP) and Global Max Pooling (GMP). The global-context features generated by these two layers are concatenated (Concat) with the multi-scale local features extracted by the serial pooling path, thereby enhancing the model’s global perception capability.

To enhance the model’s robustness in complex backgrounds and improve sensitivity to small maize seedling objects, we propose an Optimized Spatial Pyramid Pooling–Fast (OSPPF) module. The design modifies the original SPPF block (Wang et al., 2025) by appending a Global Average Pooling (GAP) layer (Jin et al., 2025) and a Global Max Pooling (GMP) layer (Ruyu et al., 2023). By combining global statistical information (GAP) with salient local responses (GMP), OSPPF provides more comprehensive feature aggregation, thereby strengthening the model’s multi-scale representation. The architecture of OSPPF is illustrated in **Figure 4**.

In the OSPPF module, GAP and GMP are inserted alongside the SPPF structure, and their outputs are concatenated. This modification effectively fuses global and local features, thereby enhancing the model’s ability to perceive targets at different scales and improving performance in complex field backgrounds with plants of varying sizes.

With these refinements, the proposed UAV-based maize seedling plant-and-leaf detection method, built upon the DP-YOLOv8 model, achieves strong performance in complex field environments and provides robust technical support for precision agricultural management. Future research will focus on extending this approach to additional crops and more challenging environments, thereby promoting the continued advancement of agricultural intelligence technologies.

#### 2.2.4 Loss function optimization strategy

To enhance the model’s ability to learn from hard samples—such as partially occluded leaves or leaves in unevenly illuminated backgrounds—we optimized the loss function by integrating a dynamic IoU compression mechanism, termed Focaler-IoU, on top of the conventional CIoU (Complete Intersection over Union) loss (Dang et al., 2024). This mechanism dynamically re-weights the loss contribution of each sample, compelling the network to focus more on difficult instances and thereby improving overall robustness.

The CIoU loss function is an extension of the traditional IoU loss that not only considers the overlapping area between the predicted and ground-truth boxes but also introduces penalty terms for shape and scale. As a result, it provides a more comprehensive measure of the discrepancy between predicted and ground-truth boxes. The formula for the CIoU loss function is given as follows:

(1) $$CIoU=1-(\frac{IoU}{1+\alpha \cdot \upsilon })$$

In Equation 1, IoU denotes the overlap-area ratio between the predicted and ground-truth boxes, α is a balancing parameter, and υ is the penalty term for shape and scale. By incorporating these penalty terms, the CIoU loss function optimizes localization accuracy more effectively.

Dynamic IoU Compression Mechanism (Focaler-IoU). Although the CIoU loss performs well in many scenarios, the model may still fail to capture sufficient features when dealing with hard samples. To further strengthen its ability to learn from difficult instances, we introduce a dynamic IoU compression mechanism, termed Focaler-IoU. This mechanism adaptively adjusts the weighting of the loss function, compelling the model to pay greater attention to hard-to-detect samples. Specifically, the CIoU loss is restructured using a linear-interval-mapping strategy, expressed as follows:

(2) $$IoU^{focaler}=\{\begin{array}{c} 0,IoU<d\\ \frac{IoU-d}{u-d},d\leq IoU\leq u\\ 1,IoU>U \end{array}$$

In Equation 2, IoUfocaler refers to the restructured dynamic IoU loss value, IoU denotes the original IoU value, and d and u are parameters within [0, 1] that control the range of dynamic adjustment. By tuning d and u, the emphasis of IoUfocaler can be shifted. Specifically, when IoU is low (indicating hard samples), the value of IoUfocaler increases, thereby raising the loss weight assigned to these difficult instances. Conversely, when IoU is high (indicating easy samples), the value of IoUfocaler decreases, reducing the loss weight assigned to these easier cases.

This dynamic adjustment mechanism enables the model to concentrate its learning on hard-to-detect samples during training, thereby improving overall robustness. In particular, when handling partially occluded leaves or leaves under uneven illumination, both detection accuracy and robustness are significantly enhanced.

#### 2.2.5 Model training and experimental parameter setting

The experiments were conducted on a workstation equipped with an NVIDIA GeForce RTX 3060 Ti GPU, 64 GB RAM, and an Intel Core i7 CPU, running Ubuntu 20.04. The deep learning framework was PyTorch 1.10 with CUDA 11.3. Model training was performed using the Adam optimizer with an initial learning rate of 0.01, momentum of 0.937, and weight decay of 0.0005. The learning-rate scheduler employed a warm-up phase followed by linear decay, instead of cosine annealing. The batch size was set to 4, with input image sizes of 8192×5460 for the plant dataset and 1024×1024 for the leaf dataset. Training was conducted for 500 epochs. To mitigate overfitting, early stopping with a patience of 50 epochs was applied, and a warm-up of 3 epochs was introduced at the start of training. Model generalization was evaluated through cross-validation and error analysis, while hyperparameters such as learning rate, optimizer, and batch size were further tuned to improve performance.

#### 2.2.6 Model performance evaluation indicators

To comprehensively evaluate the performance of the DP-YOLOv8 model on maize seedling plant and leaf detection, three primary evaluation metrics were adopted (Juntao et al., 2023): mean Average Precision (*mAP*), Precision, and Recall. These indicators jointly reflect the model’s detection accuracy and robustness from different perspectives.

Mean Average Precision (*mAP*). *mAP* is a comprehensive metric that measures detection accuracy across object categories. It is computed by first calculating the Average Precision (AP) for each class and then averaging these values. For each class, the model generates detection results with associated confidence scores. By varying the confidence threshold, a Precision–Recall curve is constructed, and AP is defined as the area under this curve, capturing performance across all confidence levels. The *mAP* is then obtained by averaging the AP of all classes, providing an overall performance indicator. A higher *mAP* indicates stronger detection accuracy across categories.

Precision measures the proportion of correctly detected objects among all objects predicted as positive by the model. It reflects the reliability of detection results, showing how many of the predicted positives are true positives. The formula is expressed as:

(3) $$Precision=\frac{TP}{TP+FP}$$

In Equation 3 TP (True Positives) denotes the number of correctly detected targets and FP (False Positives) denotes the number of incorrectly detected targets. A higher Precision value indicates more accurate detection results and a lower false-positive rate.

Recall measures the proportion of correctly detected objects relative to the total number of actual objects. It reflects the completeness of detection, showing how many of the true targets present in the dataset are successfully identified by the model. The formula is expressed as:

(4) $$\text{Recall}=\frac{\text{TP}}{\text{TP}+\text{FN}}$$

In Equation 4 TP (True Positives) denotes the number of correctly detected targets, and FN (False Negatives) denotes the number of targets that were not detected. A higher Recall value indicates that the model can detect a greater proportion of actual targets, corresponding to a lower missed-detection rate.

By jointly employing mAP, Precision, and Recall, a comprehensive evaluation of the model’s performance on maize seedling plant and leaf detection is obtained. mAP provides an overall measure of detection accuracy across categories, while Precision and Recall offer complementary perspectives on detection reliability and completeness, respectively. The combined use of these metrics enables a holistic assessment of the model, revealing both its strengths and limitations and providing a solid foundation for further optimization.

### 2.3 Software design

To address the limitations of traditional agricultural monitoring methods in complex field environments, we developed an integrated automation platform, DP-MaizeTrack. This software combines the enhanced DP-YOLOv8 model for accurate maize seedling and leaf detection, target localization, and data analysis. DP-MaizeTrack is designed to provide an efficient and user-friendly tool for agricultural professionals, enabling automated detection, real-time visualization, and precise data analysis, thereby advancing precision agriculture applications.

The software architecture of DP-MaizeTrack is modular, consisting of key components: image input and processing, object detection, result visualization, statistical analysis, and data management. Each module is clearly defined and works synergistically to ensure efficient and stable performance when processing large-scale UAV images.The image input and processing module supports the acquisition of high-resolution RGB images from various UAVs, such as the DJI Zenmuse P1. To ensure data quality, this module applies preprocessing techniques, including cropping, resizing, and background noise reduction, optimizing the input for subsequent detection. By removing redundant background elements and enhancing the features of the targets, this module ensures that the images meet the requirements of the object detection model, providing high-quality input for further analysis.The object detection module is the core component of DP-MaizeTrack, integrating the improved DP-YOLOv8 model. This model has been optimized specifically for agricultural scenarios, particularly for detecting maize seedlings and leaves in complex field environments. DP-YOLOv8 is designed to address challenges such as background clutter, plant occlusion, and lighting variation. With the incorporation of the Multi-Scale Feature Enhancement (MSFE) module and the Optimized Spatial Pyramid Pooling (OSPPF) module, the model achieves enhanced detection accuracy, especially for small objects and large-scale variations. It automatically generates bounding boxes for each detected target and outputs corresponding confidence scores, ensuring precise detection results.The result visualization module is designed to present detection outcomes in a user-friendly, graphical format. This module displays the location and class of each detected maize seedling and leaf, allowing users to quickly assess crop distribution and growth conditions. Additionally, the module supports region segmentation, generating statistical analysis reports for different regions. Through this functionality, users can better understand key data related to crop growth, aiding decision-making in agricultural production.For data management, DP-MaizeTrack automatically stores detection results, images, and analysis reports in a local database, facilitating easy retrieval of historical data. The software also supports data export in formats such as CSV and PDF, enabling users to share results with other agricultural management systems or conduct further analysis. To ensure data security and integrity, the software employs an efficient data storage structure with backup and recovery options.

To optimize performance, DP-MaizeTrack has been fine-tuned to run efficiently on standard PC configurations. The software optimizes memory usage and computational resources, enabling rapid image processing and ensuring real-time detection. It is compatible with both Windows and Linux operating systems, providing flexibility in deployment across different hardware environments.Furthermore, DP-MaizeTrack adopts a plugin-based architecture, allowing users to extend its functionality by integrating additional modules, such as crop health monitoring or pest and disease recognition. This modular design makes the software adaptable to new agricultural needs and extends its use to the detection and analysis of additional crops.

In summary, DP-MaizeTrack not only automates maize seedling and leaf detection but also provides a comprehensive data analysis platform for precision agriculture. By integrating image processing, object detection, result visualization, and data management, the software offers a powerful tool for agricultural monitoring and decision-making, contributing to the advancement of intelligent agriculture.

## 3 Results

To validate the performance of the DP-YOLOv8 model on maize seedling plant and leaf detection, a series of experiments were designed, including ablation studies and comparative evaluations. All experiments were conducted on the high-resolution UAV image dataset collected in Yuanyang County, Henan Province, under identical hardware and software environments to ensure comparability of results. Both ablation and comparative experiments were performed on maize seedling plants, with outcomes evaluated on the test set. Maize seedling leaf detection experiments are presented separately as specialized tests in Section 3.

### 3.1 Performance comparison experiments of mainstream object detection models

**Figure 5:**
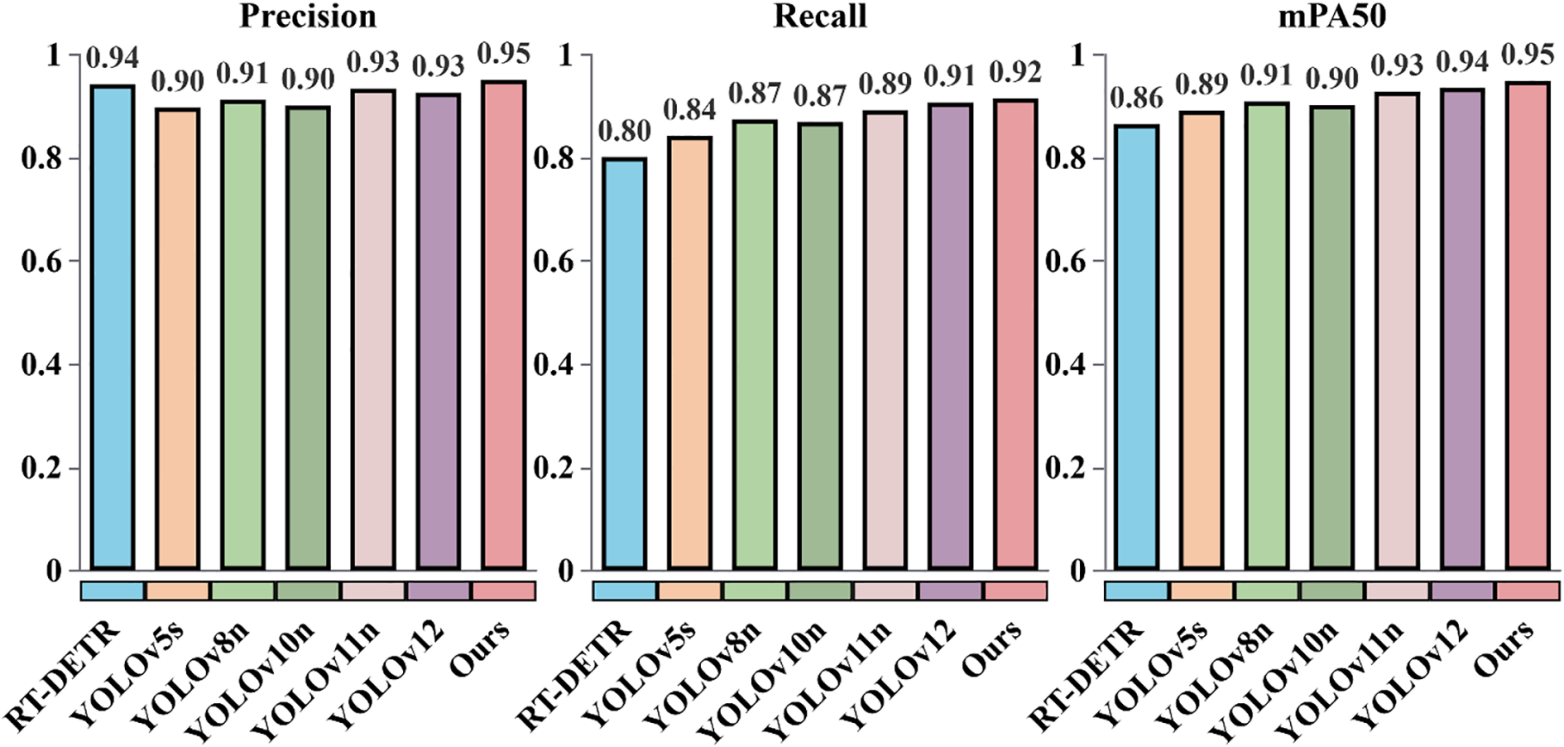
Comparative performance of the DP-YOLOv8 model and other state-of-the-art object detection models on maize seedling detection. The baseline is the original YOLOv8 model, and the improved version integrates MSFE, OSPPF, and Focaler-IoU.

To comprehensively assess the performance of the DP-YOLOv8 model on maize seedling detection, detailed comparative experiments were conducted. The improved YOLOv8 was benchmarked against several state-of-the-art object detection models, including RT-DETR (Zhou and Wei, 2025), YOLOv5 (Xia et al., 2024), YOLOv8n, YOLOv10n (An et al., 2025), YOLOv11n (Wu et al., 2025), and YOLOv12n (Ge et al., 2025). These models have all demonstrated strong detection accuracy and computational efficiency in the field of object detection. Comparing their performance provides an intuitive demonstration of the advantages of the improved YOLOv8. The results obtained on the test set are summarized in **Figure 5**, which reports Precision, Recall, and mAP50 for each model.

As shown in **Figure 5**, the DP-YOLOv8 model achieves the best overall performance among all compared models. Specifically, it reaches 94.9% in mAP50, 95.1% in Precision, and 91.5% in Recall, all of which are significantly higher than those of the other methods. Compared with the baseline model, DP-YOLOv8 improves mAP50, Precision, and Recall by 4.0, 3.9, and 4.1 percentage points, respectively. Relative to YOLOv12n, the improvements are 1.4, 2.5, and 0.8 percentage points, while compared with YOLOv11n, the gains are 2.1, 1.7, and 2.3 percentage points. In comparison with YOLOv10n, DP-YOLOv8 achieves even larger increases of 4.7, 5.0, and 4.6 percentage points in the three metrics. These results demonstrate that DP-YOLOv8 delivers substantial advantages in both detection accuracy and robustness, enabling more precise recognition and localization of maize seedlings.

### 3.2 Ablation analysis of the model improvement module

Ablation experiments were conducted to isolate and evaluate the individual contributions of each proposed component. The modules—MSFE, OSPPF, and Focaler-IoU—were incorporated incrementally. To quantify their specific effects, each module was tested independently as well as in all possible combinations. The corresponding results on the test set are summarized in **Table 1**.

**Table 1:** Results of the ablation study.

Model	Precision	Recall	mAP50
YOLOv8n (baseline)	0.912	0.874	0.909
YOLOv8n-MSFE	0.939	0.906	0.94
YOLOv8n-MSFE+OSPPF	0.946	0.942	0.942
Ours	**0.951**	0.915	**0.949**

Baseline denotes the original YOLOv8; +MSFE refers to the insertion of the Multi-Scale Feature Enhancement module into the Backbone; +OSPPF replaces the original SPPF with the Optimized Spatial Pyramid Pooling–Fast module; and +Focaler-IoU substitutes the CIoU loss with the Focaler-IoU loss. Some bolded indicators indicate that in the improved model, these indicators are the best among all the comparison models.

As shown in **Table 1**, each proposed module contributes to a clear performance improvement. MSFE markedly enhances the model’s perception of multi-scale targets, particularly small objects and large-scale variations. With MSFE alone, Precision increases from 0.912 to 0.939, Recall from 0.874 to 0.906, and mAP50 from 0.909 to 0.940, underscoring its pivotal role in multi-scale perception. Building on MSFE, the addition of OSPPF further improves performance: Precision rises from 0.939 to 0.946, Recall from 0.906 to 0.942, and mAP50 from 0.940 to 0.942, indicating that OSPPF strengthens the model’s adaptation to complex backgrounds, especially under background clutter or plant-size variations. Finally, incorporating Focaler-IoU on top of MSFE + OSPPF yields the best results: Precision increases from 0.946 to 0.951, Recall from 0.942 to 0.915, and mAP50 from 0.942 to 0.949. This demonstrates that Focaler-IoU significantly enhances the model’s ability to learn from hard samples and improves robustness, particularly for partially occluded leaves and leaves under uneven illumination.

Overall, the ablation study clearly delineates the role of each module: MSFE enhances multi-scale perception, OSPPF strengthens adaptation to complex backgrounds, and Focaler-IoU facilitates learning from hard samples. Their synergistic integration enables the improved YOLOv8 to substantially outperform the baseline and competing models on maize seedling detection tasks.

**Figure 6:**
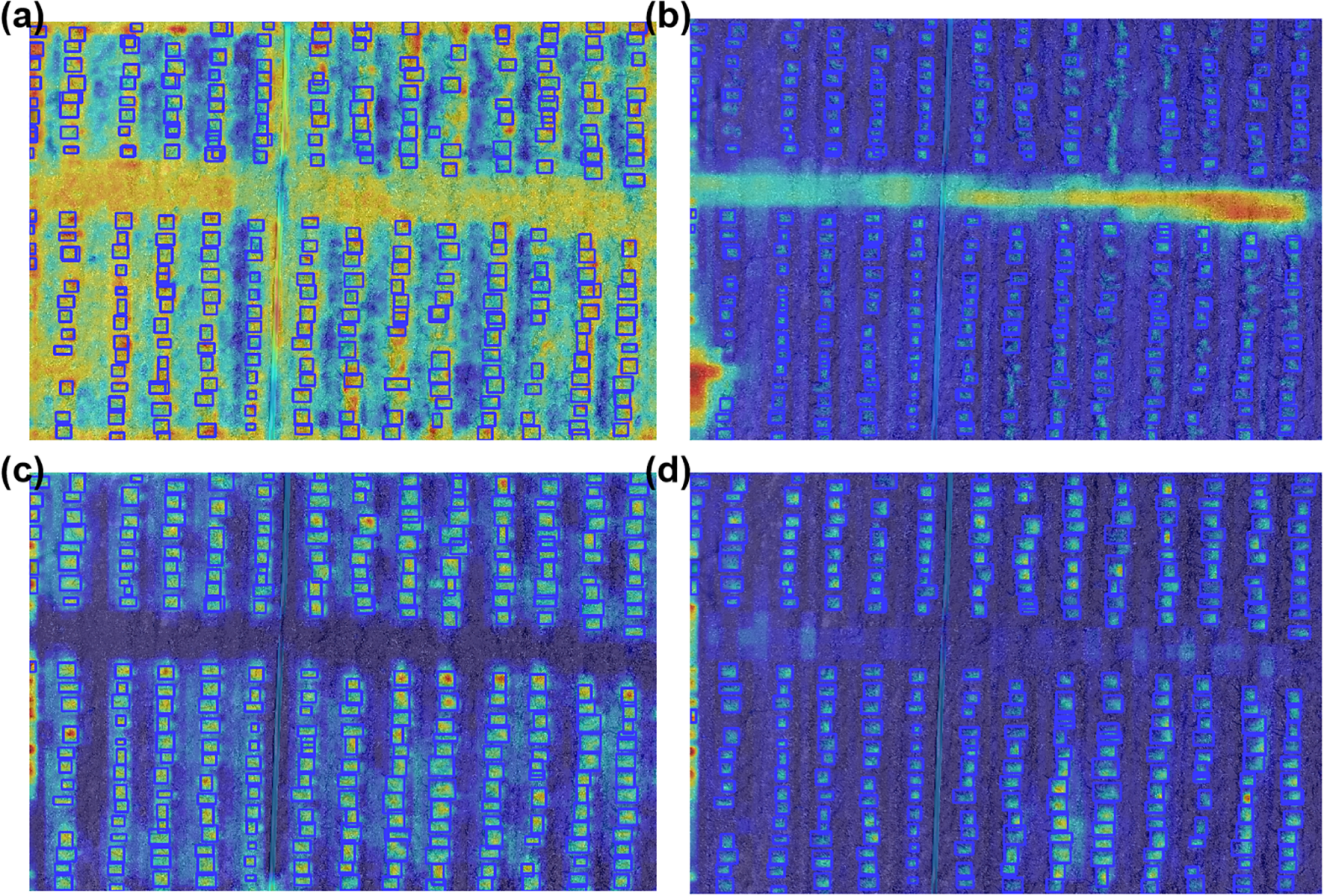
Visualization results of the ablation experiments. **(a)** Baseline YOLOv8 model; **(b)** YOLOv8 with the MSFE module; **(c)** YOLOv8 with the MSFE module and optimized SPPF; **(d)** DP-YOLOv8 with the MSFE module, optimized SPPF, and improved loss function.

To further analyze the specific role of each improved module and to present their effects more intuitively, Grad-CAM was employed for the visualization of the ablation experiments. This approach enables a clear illustration of how different model variants behave in complex field environments and reveals the evolution of detection performance as new modules are introduced. Compared with relying solely on quantitative metrics, Grad-CAM–based visualizations directly reflect the stability of bounding box distributions, the accuracy of target localization, and the suppression of background interference, thereby providing stronger empirical support for the effectiveness of each improvement. The visualization results are shown in **Figure 6**.

As illustrated in **Figure 6**, the baseline YOLOv8 model exhibits notable limitations under complex field conditions, characterized by scattered bounding boxes, insufficient localization accuracy of maize seedlings, and a high false detection rate in regions with dense weeds or complex soil textures. This indicates that the baseline model struggles with multi-scale feature extraction, with its receptive field unable to effectively distinguish subtle differences between target objects and background noise. After incorporating the MSFE module, the model’s ability to perceive multi-scale features improves substantially, resulting in tighter bounding box fitting and stronger responses in seedling regions. Nevertheless, background interference remains evident near field edges and in non-plant areas, suggesting that MSFE alone cannot fully suppress environmental noise. When combined with the optimized SPPF, the model demonstrates stronger robustness in feature fusion and spatial information aggregation, further enhancing detection consistency and stability, though slight noise can still be observed in highly complex regions. Finally, with the integration of MSFE, the optimized SPPF, and the Focaler-IoU loss function, the complete DP-YOLOv8 model achieves significant improvements: it consistently captures seedling features across multiple scales, effectively suppresses background interference, and produces bounding boxes that are uniformly distributed and highly aligned with the actual positions of maize seedlings. These comparative results clearly validate the progressive contributions and synergistic effects of the proposed modules, demonstrating the effectiveness and robustness of DP-YOLOv8 for high-precision detection in complex field environments.

### 3.3 Special experiment on corn leaf detection

To further validate the model’s performance on a specialized task and to support subsequent leaf-age analysis, a dedicated maize leaf-tip detection experiment was conducted. Leaf-tip detection poses unique challenges—such as strong background interference and pronounced morphological variation—making this task considerably more difficult (Zhou et al., 2025). The improved model was benchmarked against the state-of-the-art YOLOv12 model for leaf detection and additionally compared with single-plant detection. The corresponding results are summarized in **Table 2**.

**Table 2:** Comparison of leaf-level and single-plant detection.

Model	Precision	Recall	mAP50
YOLOv12 (leaf)	0.581	0.577	0.581
YOLOv8 (leaf)	0.647	0.551	0.517
Ours (leaf)	0.638	0.519	0.528
YOLOv12 (plant)	0.926	0.907	0.935
YOLOv8n (plant)	0.912	0.874	0.909
Ours (plant)	**0.951**	**0.915**	**0.949**

YOLOv12, the baseline YOLOv8, and the improved YOLOv8 were applied to both leaf-tip and single-plant detection. The table reports each model’s performance on the two tasks and highlights the relative effectiveness of leaf-level versus single-plant detection. Some bolded indicators indicate that in the improved model, these indicators are the best among all the comparison models.

As shown in **Table 2**, the results indicate that in the single-plant detection task, the improved model consistently outperforms the alternatives, achieving 0.951 Precision, 0.915 Recall, and 0.949 mAP50. These represent gains of 3.9, 4.1, and 4.0 percentage points over the baseline YOLOv8n, and also surpass YOLOv12’s corresponding scores of 0.926, 0.907, and 0.935. By contrast, all models exhibit a substantial performance decline on the leaf-tip detection task. The improved model reaches 0.638 Precision, 0.519 Recall, and 0.528 mAP50, slightly exceeding YOLOv12’s values of 0.581, 0.577, and 0.581 in Precision, but remaining far below the plant-level results. This highlights that detecting small objects such as leaf tips remains highly challenging and requires further optimization. The performance gap primarily arises from the irregularity of leaf annotations: the training set labels only the leaf tips, whose variable lengths and widths lack a consistent standard, thereby reducing training effectiveness.

### 3.4 Development and application of software platform for corn seedling plant and leaf detection

To enable deployment of the DP-YOLOv8 model in real-world agricultural monitoring, an integrated software platform was developed that unifies maize seedling plant detection and leaf detection within a user-friendly interface. The platform provides agricultural researchers and practitioners with an efficient tool for rapid assessment and analysis of maize seedling growth, delivering foundational data for subsequent leaf-age analysis experiments.

**Figure 7:**
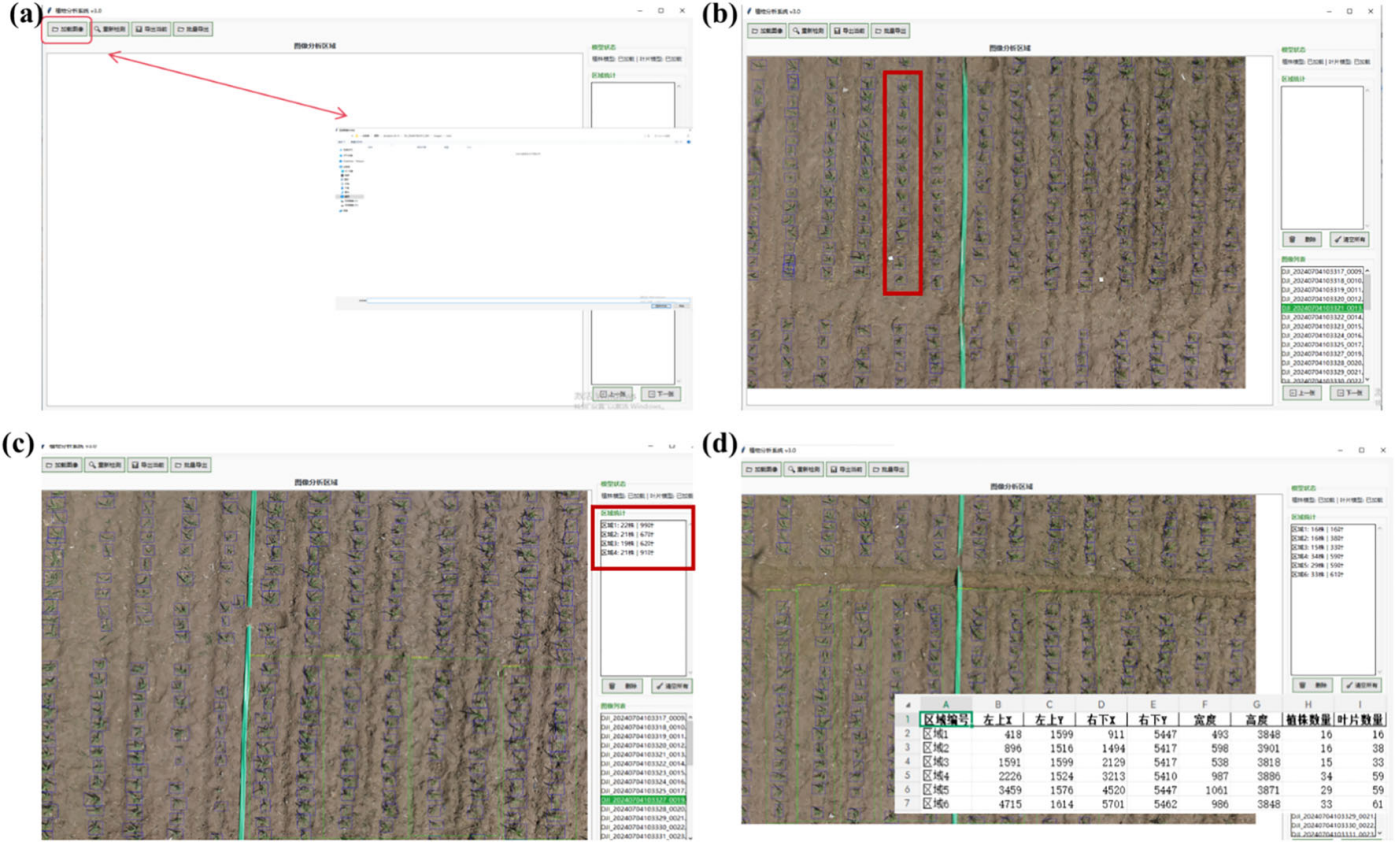
Main functional modules of the maize seedling analysis platform. **(a)** Data import and preprocessing: supports the import of high-resolution UAV images and provides basic operations such as cropping and preprocessing; **(b)** Plot segmentation and automatic ID assignment: divides field images into plots and automatically assigns a unique identifier to each plot for subsequent statistical analysis; **(c)** Seedling and leaf detection with counting: automatically detects maize seedlings and their leaves within user-selected plots and visualizes the results with bounding boxes and labels; **(d)** Data export and statistical reporting: exports detection results and plot-level statistics to Excel for further analysis and record-keeping.

The platform offers multiple functions, including image import and preprocessing, plot segmentation and detection, result visualization, and data export. Users can import high-resolution UAV images and perform preprocessing operations such as plot segmentation, plant detection, and leaf detection. The software detects and counts maize seedling plants and leaves within user-selected regions, with detection results displayed in an intuitive graphical interface that clearly shows bounding boxes and class labels. Results can be exported to Excel for further analysis and record-keeping. In addition, the platform provides basic data-analysis functions, such as counting the number of plants and leaves, thereby offering essential data support for subsequent leaf-age analysis (Yang et al., 2025). The software interface is shown in **Figure 7**.

**Figure 7** illustrates the main interface of the software. Through the navigation bar, users can access different functional modules. The interface consists of two primary sections: the left-hand image-display area, where images can be viewed and manipulated, and the right-hand control panel. In the image-display area, users can select a specific region (plot) by clicking and dragging; the software then detects and counts maize seedling plants and leaves within that region. The control panel includes modules for model status, image control, region statistics, image list, and data export. Using the control panel, users can load images, rerun detection, delete or clear regions, and export detection results.

**Figure 8:**
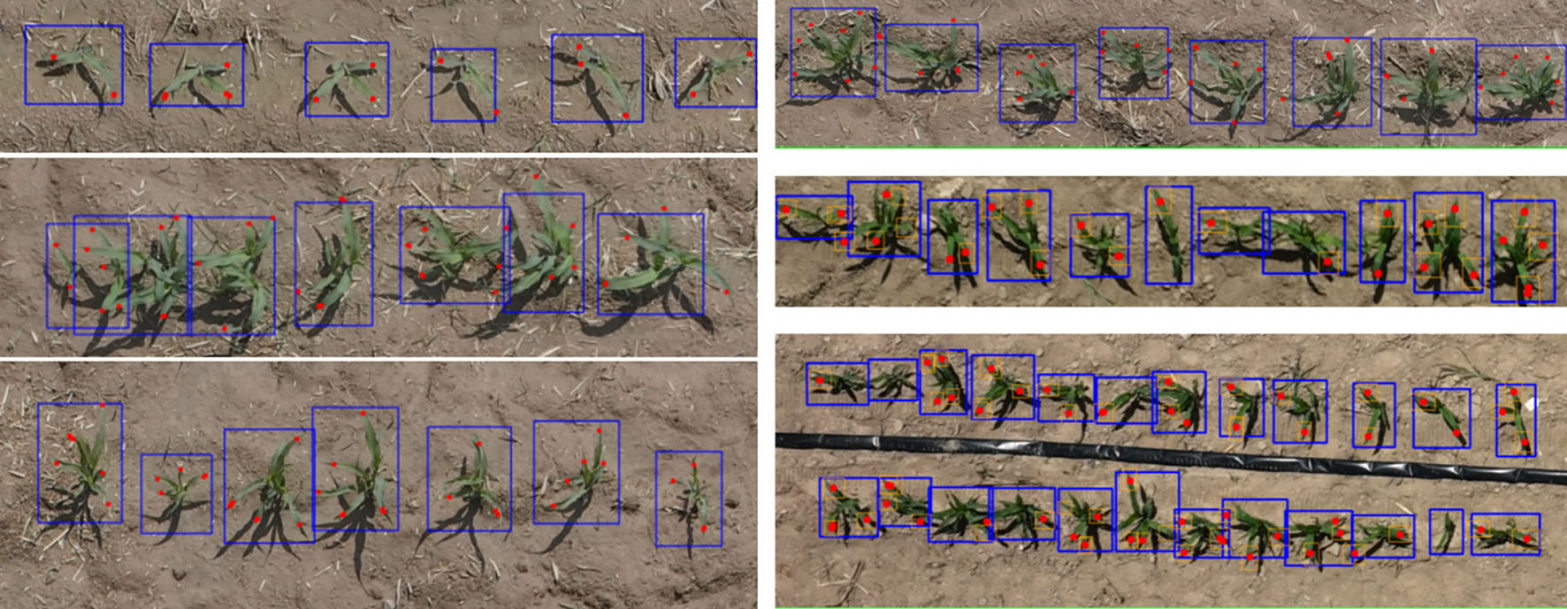
Visualization of maize seedling detection results across the 2-leaf to 6-leaf stages. Blue bounding boxes indicate individual maize seedlings, and red dots mark the detected leaf tips.

The software platform was developed to bring state-of-the-art object detection into practical agricultural scenarios, thereby providing robust support for precision farm management. By integrating the DP-YOLOv8 model, the application enables rapid and accurate detection of both maize seedlings and their individual leaves. The resulting visualizations are shown in **Figure 8**.

Following the visualization results, a user validation study was conducted to evaluate the quantitative performance of the software in practical applications. The seedling and leaf counting outputs generated by the platform were compared with manually annotated ground-truth data. As shown in **Table 3**, seedling counting maintained consistently high accuracy across different growth stages, with values exceeding 96%. In contrast, leaf counting accuracy gradually decreased as the number of leaves increased, from approximately 85.0% at the 2-leaf stage to about 76.0% at the 8-leaf stage. This indicates that the platform provides stable and reliable performance at the seedling level, whereas leaf-level counting is more affected by factors such as occlusion, overlap, and variations in leaf morphology. Overall, although leaf detection remains a challenging task, the high accuracy of seedling detection and the overall reliability of the platform have been validated, confirming its effectiveness in supporting real-time field monitoring and leaf-age analysis.

**Table 3:** Accuracy of maize seedling and leaf counting using the analysis platform.

Growth stage	Seedling counting accuracy (%)	Leaf counting accuracy (%)
2-leaf	98.1	85.0
4-leaf	97.6	82.0
6-leaf	97.1	79.0
8-leaf	96.4	76.0
**Average**	**97.3**	**80.5**

Seedling detection maintained consistently high accuracy (>96%) across growth stages, while leaf counting accuracy declined from about 85% at the 2-leaf stage to 76% at the 8-leaf stage, reflecting the increased challenges caused by occlusion and overlap. Some bolded indicators indicate that in the improved model, these indicators are the best among all the comparison models.

By leveraging the platform’s semi-automated workflow, growers can rapidly segment field plots, count individual maize seedlings and their leaves during the seedling stage, and monitor crop status in real time—thereby enabling timely, data-driven decision-making. With its intuitive interface and high efficiency, the platform represents a valuable tool for agricultural monitoring and management.

## 4 Discussion

The DP-YOLOv8 model demonstrates significant detection accuracy and robustness across diverse and complex field environments. In the maize seedling single-plant detection task, it achieves 95.1% Precision, 91.5% Recall, and 94.9% mAP. Compared with the baseline YOLOv8n, Precision increases by 3.9%, Recall by 4.1%, and mAP by 4.0%. Compared with the latest YOLOv12, Precision increases by 2.5%, Recall by 0.8%, and mAP by 1.4%. These results indicate that the proposed model outperforms existing methods in both accuracy and stability, showing stronger reliability under challenging conditions such as complex backgrounds, uneven illumination, and partial occlusion. Based on this model, the DP-MaizeTrack software platform transforms high-precision detection results into intuitive visualizations and statistical information. Users can not only obtain single-plant level recognition but also monitor the number of seedlings and leaves across larger field regions, enabling the research outcomes to directly serve field management and breeding practices.

From a methodological perspective, the proposed improvements introduce clear technical innovations and provide solutions to multiple challenges in field environments. The MSFE module enhances multi-scale perception by combining the ECA attention mechanism with multi-branch feature extraction, effectively addressing differences in seedling scale at various growth stages and imaging heights. The ECA mechanism dynamically adjusts channel weights before feature extraction, highlighting relevant features while suppressing redundant information, whereas the multi-branch structure with different receptive fields captures both fine-grained and large-scale features, maintaining detection stability under significant scale variations. The OSPPF module strengthens adaptability to complex backgrounds. In field scenarios where soil textures, weeds, and shadows may easily confuse the model, OSPPF introduces Global Average Pooling (GAP) and Global Max Pooling (GMP) into the traditional SPPF structure and fuses them with local features. GAP extracts overall semantic information to reduce false detections, while GMP emphasizes prominent local regions, together forming a more robust feature representation. Meanwhile, the Focaler-IoU loss function improves the model’s ability to learn from hard samples. By dynamically re-weighting the loss, it directs attention to partially occluded or poorly illuminated samples, preventing them from being overlooked during training and thereby enhancing robustness under challenging conditions. Overall, these three improvements address the issues of large scale variation, complex background interference, and insufficient learning of hard samples, resulting in a detection framework better suited for real field applications. At the same time, the DP-MaizeTrack software platform integrates these model improvements into an automated pipeline for detection, segmentation, and statistical analysis, ensuring that high-accuracy results can be directly utilized without requiring complex programming, thereby increasing the practicality and applicability of the system.

Despite these advances, certain limitations remain. While the model performs strongly at the seedling level, leaf-level detection shows considerably lower Precision, Recall, and mAP, revealing limitations in fine-grained feature extraction. Leaf tips, in particular, exhibit substantial morphological variability, slender and irregular structures, and are more susceptible to background interference and uneven illumination, which often lead to false detections and missed detections. Another possible reason is insufficient effective learning of leaf data during training. Although the dataset contains many leaf annotations, the labeling focuses only on leaf tips, which are variable in size, irregular in shape, and subject to strong subjectivity. The lack of a unified annotation standard reduces stability during feature learning and weakens the model’s generalization ability for leaf detection. Although the MSFE and OSPPF modules are effective for seedling detection, they do not fully capture the fine-grained features required at the leaf level, further exposing the limitations of the current method. Correspondingly, the DP-MaizeTrack platform, which relies on the model output for leaf-level visualization and statistical analysis, is also constrained in accuracy and stability, highlighting the need for further improvements to the leaf-detection module.

Future research should proceed along two main directions. On the model side, increasing the diversity and volume of leaf annotations, refining annotation strategies to reduce subjectivity in scale and orientation, and adopting network architectures and instance segmentation methods tailored (Yu et al., 2024) to fine-grained detection are expected to improve accuracy and robustness for leaf detection. On the software side, the DP-MaizeTrack platform can be further expanded to incorporate additional functions, such as dynamic growth monitoring based on temporal UAV imagery or integration with agricultural IoT systems to enable automated seedling assessment and intelligent decision support. Through the dual advancement of model optimization and software extension, the proposed framework has the potential to be widely applied to crop monitoring and precision agriculture management, thereby contributing to the development of smart and efficient agricultural systems.

## 5 Conclusions

This study develops DP-MaizeTrack, a UAV-based intelligent detection and visualization platform for maize seedlings and leaves, which integrates the improved detection model DP-YOLOv8 as its core. The model incorporates a Multi-Scale Feature Enhancement (MSFE) module and an Optimized Spatial Pyramid Pooling–Fast (OSPPF) module into the backbone, together with a dynamic IoU-compression loss function (Focaler-IoU). Experiments on a field-collected dataset demonstrate that DP-YOLOv8 significantly outperforms both the baseline YOLOv8n and the latest YOLOv12, achieving 95.1% Precision, 91.5% Recall, and 94.9% mAP50, thereby improving robustness under complex backgrounds, uneven illumination, and plant occlusion. The DP-MaizeTrack platform further integrates region segmentation, statistical analysis, and visualization functions, enabling high-accuracy detection results to be transformed into actionable agricultural information, and providing practical support for precision crop management and breeding research. Although challenges remain in leaf-level detection, particularly for extremely small targets such as leaf tips (with Precision, Recall, and mAP50 of 63.8%, 51.9%, and 52.8%, respectively), the overall findings confirm the effectiveness of DP-MaizeTrack in handling multi-scale targets and cluttered field environments. This work lays a solid foundation for subsequent leaf-age analysis, population-structure modeling, and cross-crop extension, and highlights the potential of UAV-based deep learning software platforms to advance intelligent and data-driven agricultural monitoring.

## Data Availability

The data analyzed in this study is subject to the following licenses/restrictions: Unauthorized reproduction or distribution in any form, in whole or in part, is prohibited, including uploading to public platforms, sharing with third parties, or using as datasets for public competitions or evaluations. Requests to access these datasets should be directed to 2504388813@qq.com.
